# Rambles in Military Hospitals

**Published:** 1864-07

**Authors:** R. M. Lackey

**Affiliations:** M. D., A. A. Surg., U. S. A.; Rock Island Barracks, Ill.


### RAMBLES IN MILITARY HOSPITALS.

REPORT OF

### A CASE OF ENCYSTED ABSCESS OF THE BRAIN.

By R. M. LACKEY, M. D., A. A. Surg., U. S. A.

James E. Stephenson, prisoner of war, aged 22 years, sanguine temperament, and apparently of good constitution, was attacked with small-pox, Jan. 12th, 1864. He had the distinct form of the disease, and so light that he remained only ten days at the Pest Hospital. Immediately after his return to barracks from small-pox hospital, Jan. 22d, he began complaining of constant and severe pain in the right supra-orbital and temporal regions, attended with some febrile action during the first few days. Nothing serious was suspected by the surgeon under whose care he was, and he was allowed to remain in barracks until March 22d, when he was sent to hospital. At the time of admission he still had the constant pain in the regions named, very slow pulse, 40 to 50, cool skin, bowels constipated, and but little appetite.

Various kinds of treatment were employed, but without affording permanent relief. He was kept upon tonics, however, and occasionally an anodyne was given hypodermically. Subsequent to his admission to hospital, he had a discharge of matter from the ear, that continued two or three weeks when it ceased. His mental faculties became gradually obtuse, and he remained on his cot nearly all the time, with his eyes closed as if asleep; but he could be easily aroused, and would say that he had not been asleep, and would answer questions and talk slowly but correctly. He contended all the time that there was "some round substance inside of his head" that would roll around and change position as he turned his head from one side to the other, and so painful was this sensation, that he would sometime remain for hours without moving his head from the pillow.

About the 3d of April, a fluctuating tumor appeared behind the ear, which was opened, and pus discharged for several days. After this abscess was opened the patient was much relieved, became more lively, walked around the ward, and conversed freely with the attendants, and seemed in a fair way to recover. As soon, however, as the pus ceased discharging, the pain and other symptoms gradually appeared again, and grew worse until May 1st, when he was taken with convulsions, which increased in severity and frequency, and he died on the 3d.

There was no paralysis, or abnormal condition of the pupils of the eyes, up to the time he was seized with convulsions, but after the first paroxysm, the pupil of the *right* eye was dilated —and paralysis, such as to render articulation and deglutition difficult. During the interval between the paroxysms he remained comatose, and it was with difficulty that he could be aroused sufficiently to answer questions.

Post-mortem examination was made 12 hours after death. Drs. Morrison, Nesbit, and others were present. The brain was the only part examined. No abnormal appearance was presented on removing the calvarium, and the dura mater from the upper surface of the cerebrum. While removing the brain, and on making slight pressure on the right middle lobe, a large quantity of greenish looking pus gushed out. The organ was removed entire, and on examination an opening, from which the pus escaped, was found in the middle lobe on the right side, at that portion corresponding to a point on the skull where the anterior surface of the petrous joins the squamous portion of the temporal bone—immediately behind the base of the zygomatic process. Portions of the brain were sliced off, and the right middle lobe was found to be nearly all destroyed by the abscess; the pus was contained in a cyst that was so thick and strong that I had no difficulty in separating it from the surrounding substance, and preserving it without any break, except the opening from which the pus escaped whilst removing the brain. This cyst will hold about four ounces of fluid. The substance of the brain surrounding the cyst was not in the least softened, and seemed to be entirely healthy. The ventricles were nearly full of a dirty looking fluid. The other parts of the organ presented, to the naked eye, nothing abnormal.

On examining the skull at the point designated above, an oval opening was discovered in the bone and dura mater, one-half inch in diameter. A portion of the skull, including the diseased part, was removed and preserved. The bone immediately around the opening is not much carious—indeed, there is so little appearance of disease that one would almost think the hole had been made with a drill.

This, then, seems to have been a case of *encysted abscess of the brain*, communicating externally through the opening in the bone and dura mater; and the matter producing the abscess behind the ear, was undoubtedly formed internally. The fluctuating tumor behind the ear appeared suddenly, and on opening it, the pus found to be beneath the periosteum, which on examination after death was found separated from the bone at that point. The escape of pus from the abscess in the brain, was stopped by the closure of the opening in the bone, produced by the thickening of the tissues externally, which was also proven to be the case by post-mortem examination.

This man had never, as we could learn, had any injury of the head sufficient to produce the disease, and he had never complained of pain in the head before having small-pox. He had never had syphilis, and had no indications of scrofulous diathesis.

Rock Island Barracks, Ill., May 20th, 1864.

